# Design and Preliminary Evaluation of a Tongue-Operated Exoskeleton System for Upper Limb Rehabilitation

**DOI:** 10.3390/ijerph18168708

**Published:** 2021-08-18

**Authors:** Zhenxuan Zhang, Boris I. Prilutsky, Andrew J. Butler, Minoru Shinohara, Maysam Ghovanloo

**Affiliations:** 1 School of Electrical & Computer Engineering, Georgia Institute of Technology, Atlanta, GA 30308, USA; zhenxuanjameszhang@gmail.com; 2 School of Biological Sciences, Georgia Institute of Technology, Atlanta, GA 30332, USA; shinohara@gatech.edu; 3 School of Health Professions, The University of Alabama at Birmingham, Birmingham, AL 35294, USA; andrewbutler@uab.edu; 4 Bionic Sciences Inc., Atlanta, GA 30316, USA; mghovan@gmail.com

**Keywords:** tongue drive system, KINARM, stroke, robotic rehabilitation, exoskeleton, hemiplegia

## Abstract

Stroke is a devastating condition that may cause upper limb paralysis. Robotic rehabilitation with self-initiated and assisted movements is a promising technology that could help restore upper limb function. Previous studies have established that the tongue motion can be used to communicate human intent and control a rehabilitation robot/assistive device. The goal of this study was to evaluate a tongue-operated exoskeleton system (TDS-KA), which we have developed for upper limb rehabilitation. We adopted a tongue-operated assistive technology, called the tongue drive system (TDS), and interfaced it with the exoskeleton KINARM. We also developed arm reaching and tracking tasks, controlled by different tongue operation modes, for training and evaluation of arm motor function. Arm reaching and tracking tasks were tested in 10 healthy participants (seven males and three females, 23–60 years) and two female stroke survivors with upper extremity impairment (32 and 58 years). All healthy and two stroke participants successfully performed the tasks. One stroke subject demonstrated a clinically significant improvement in Fugl-Meyer upper extremity score after practicing the tasks in six 3-h sessions. We conclude that the TDS-KA system can accurately translate tongue commands to exoskeleton arm movements, quantify the function of the arm, and perform rehabilitation training.

## 1. Introduction

Stroke is the leading cause of adult disability in the United States. Of all the stroke survivors, around 80% experience different degrees of upper limb paresis, which reduces their quality of life severely [1,2]. Rehabilitation can help stroke survivors reduce disability and regain their independence [2,3]. Extensive research has identified the most effective strategies for stroke rehabilitation ranging from movement therapy to complementary medicine [4,5]. Among promising rehabilitation strategies, robot-assisted rehabilitation has been tested for its ability to improve recovery and lower the cost of stroke rehabilitation [6,7,8]. Although it is still not clear if robotic rehabilitation can deliver consistently better clinical outcomes compared to traditional therapy [7,8,9,10], robotic rehabilitation enables clinicians to deliver more consistent therapy with measurable results in real-time [11,12] with potentially lower costs [6].

While robotic rehabilitation involving passive arm movement may provide some clinical benefits [13], the fact that such interventions have little effects on motor control outcome suggests that passive movements assisted by a robot alone are not sufficient, and active participation from participants may bring better clinical outcomes [14]. Robotic rehabilitation that requires voluntary effort and provides constant challenge to a person’s motor ability by adapting to the progressive motor function improvements offers better clinical outcome compared to passive robotic training [15]. These results are consistent with the current understanding of the neurobiology of recovery after neurological injury [16,17,18] as well as with the current trends in robot-assisted upper-limb stroke rehabilitation [12].

Several human-computer interaction-based methods have been considered to harness one’s voluntary effort by detecting user intent and providing voluntary control to a rehabilitation robot. These methods include triggering and providing robot assistance based on a simple preset timer, mechanical variables (e.g., force or velocity) of the impaired or less-impaired limb, electromyographic (EMG) or electroencephalographic (EEG) signals or gaze tracking. However, these methods have limitations. Intent detection and providing voluntary control based on impaired limb movement is the most intuitive way to control a robot. However, approximately 30% of stroke survivors have severe upper extremity paresis [1], these people have difficulties performing movement with their affected upper limb [14]. In theory, using the less-impaired limb for intent detection and providing voluntary control of the rehabilitation robot could promote functional recovery of the impaired limb through coupling effects [19]. However, a Cochrane review reported that there was no significant improvement of paretic arm function with bilateral arm training using this method compared with the usual care following a stroke [20]. This result suggests that using the less-impaired limb to provide user intent to and control of a robot might not produce a positive clinical outcome. While EMG can capture electrical activity produced by skeletal muscles, the EMG pattern recognition approach might not be a practical modality to decode movement intention of stroke survivors [21]. EEG-based brain-machine interface (BMI) methods have shown promise in restoring upper extremity motor function in stroke survivors [22,23]. However, the EEG-based BMI may be difficult to use in a rehabilitation environment due to the considerable amount of time and effort to setup and train an individual to use it [24]. A recent study [25] has shown that gaze tracking can be used to capture the movement intention of healthy volunteers. Although much faster involuntary components of gaze movement and control, such as gaze shifts [26], would make the use of gaze tracking for voluntary robotic control difficult.

We have demonstrated that tongue motion, if properly harnessed, can be used to communicate human intent and to assist in controlling a rehabilitation robot or an assistive device [27,28]. The tongue has several advantages compared with the other methods of intention detection and control of rehabilitation devices. The tongue has a strong representation in the human motor cortex, a direct connection to the brain through cranial nerves, and numerous inherent and intuitive capabilities that can be employed to overcome the limitations discussed above [29,30]. The tongue can also move rapidly and accurately in almost any direction within the oral space without training. Access to the tongue is readily available noninvasively and its muscle fibers are fatigue-resistant, allowing usage of a tongue-operated rehabilitation system over extended periods of time [31]. Although speech and swallowing are often affected by a stroke [32,33,34], acute and chronic stroke survivors generally maintain their voluntary tongue control that allows them to perform tongue resistance training and improve tongue control and strength [35,36]. Therefore, the tongue is a potential means for controlling robotic rehabilitation devices with one’s own intention and effort. Another important observation is that the topographical alterations of the sensorimotor cortex can shift the motor representation of the tongue into the cortical region of the hand representation in people with cervical SCI [37] and congenital absence of one arm [38] due to the close proximity of the tongue and arm representations. Therefore, by engaging both tongue and upper limbs in synchrony, their representations in the primary motor cortex may reorganize and upper extremity function may improve, thanks to brain neuroplasticity. In our preliminary study of healthy volunteers, we observed a greater EEG signal desynchronization over the somatosensory cortex when tongue protrusions and wrist extensions were performed synchronously compared to separate executions of these movements [39]. This greater desynchronization implies facilitation of brain excitability for limb movement, which may potentially contribute to enhanced rehabilitation outcome in stroke survivors. There is another potential benefit of using the tongue for voluntary control of robot-assisted arm movements in stroke rehabilitation. It is related to the fact that general features of arm movements, e.g., letter forms in handwriting [40] or the relationship between the movement time and movement difficulty (Fitts’ law) in reaching [41], do not depend on a specific motor effector (arm, leg, head, trunk, eyes) and are generated by higher cortical levels of motor control hierarchy [42]. Therefore, using the tongue for executing reaching and tracking tasks can potentially strengthen damaged cortical sensorimotor pathways responsible for the above general invariant features of voluntary movements.

In a previous study, a tongue-operated rehabilitation robot has been developed to translate tongue motion to commands via the tongue drive system (TDS) [43,44,45,46]. Commands were used to control a wrist-based rehabilitation robot called the hand mentor [27,47]. This device has been shown to elicit improvements in strength and range of motion in moderate to severely impaired stroke survivors [28,48].

However, the aforementioned study had several shortcomings that could potentially limit clinical outcomes. The hand mentor contains only one pneumatic pump that operates one degree-of-freedom (DoF). In addition, it is controlled by an on/off discrete signal. As a result, the robot produces assistive force in only one direction (wrist extension). Due to the on/off switch control, natural and proportionally graded hand movements are not possible.

Given the potential benefits of a tongue-controlled robot-assisted rehabilitation and the limitations of the current tongue-controlled assistive robot Hand Mentor, the goal of this work was to evaluate a novel tongue-operated upper extremity robotic rehabilitation system (TDS-KA), which we have developed, that integrates the TDS and a commercially-available bimanual upper extremity exoskeleton KINARM (BIKIN Technologies, Kingston, ON, Canada). An advantage of the KINARM and other similar commercially available arm rehabilitation robots, e.g., InMotion (BIONIC, Toronto, ON, Canada), Reo Go (Motorika, Caesarea, Israel), and Armeo Spring (Volketswil, Switzerland), over the hand mentor is that the KINARM can support the weight of the arm and provides movements with two DoF (shoulder and elbow flexion and extension) in a horizontal plane. Here, we present the design of the TDS-KA system and preliminary results of its use. We demonstrate the functionality and feasibility of the system using two custom developed tasks with different control modes. We tested these tasks in 10 healthy participants. In addition, we tested a suitable rehabilitation protocol in two stroke survivors.

A preliminary version of this work has been reported in the American Congress of Rehabilitation Medicine 2017 [49].

## 2. Materials and Methods

### 2.1. System Description 

The TDS system is used to convert tongue motion to either discrete commands (rest, left, right, up or down) [46] or proportional commands (a continuous number from −1 to 1) [50]. These commands are fed into KINARM to control the exoskeleton to complete rehabilitation tasks accordingly.

**Figure 1:**
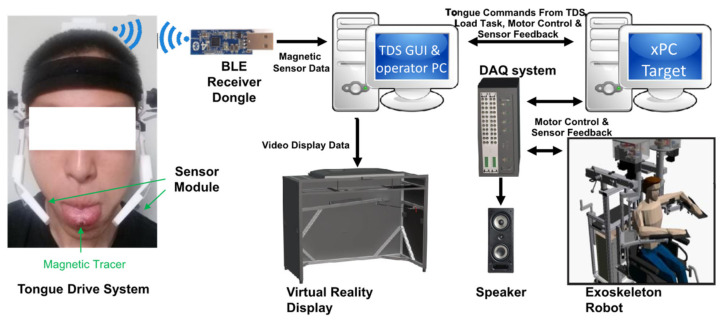
Functional block diagram of the TDS-KA system. Tongue movements are captured using a magnetic tracer on the tongue and a headset with magnetic sensors. The magnetic sensor data corresponding to the tongue position are transferred to a LabVIEW based graphical user interface (GUI) on a PC via Bluetooth low energy connection. The magnetic sensor data are further converted to either discrete commands (rest, left, right, up or down) [45] or proportional commands (a continuous number from −1 to 1) to control the exoskeleton robot via xPC target computer. At the same time, the robot operator PC controls a virtual reality display directly and the robot via a xPC target computer. The xPC target computer interacts with the exoskeleton robot via a data acquisition board (DAQ) and generates the sound queue of the task via a connected speaker. The subject’s image is published with written informed consent.

The TDS consists of a disk-shaped magnetic tracer (D21B-N52, K&J Magnetics, Inc., Jamison, PA, USA), a headset with magnetic sensors and transmitter, and a Windows-based PC with an attached USB receiver dongle (Figure 1; written informed consent was obtained from the subject to publish the image). The magnetic tracer is attached ~1 cm posterior to the tip of the participant’s tongue via tissue adhesive (Vetbond 1469Sb, 3M, Maplewood, MN, USA). A thin layer of this n-butyl-cyanoacrylate-based adhesive is applied to a small (~5 × 5 mm) dried surface of the tongue and keeps the magnetic tracer in place for several hours. Subsequently, the tracer peels off without affecting the tongue mucosa or causing undo pain, as shown previously [45,48].

A LabVIEW (National Instruments Corp., Austin, TX, USA) based graphical user interface was developed to control the TDS. Prior to TDS use, an external magnetic field (EMF) attenuation procedure was performed. Subsequently, a pattern recognition support-vector machine-based algorithm with 93% classification accuracy [51] was trained to map tongue gestures and their corresponding magnetic flux density fields captured by sensors to discrete or continuous commands. The tongue commands were sampled by KINARM at 200 Hz.

Compared to the previously published description of the system [46], we made a number of enhancements that makes TDS more robust. We developed a preprocessing algorithm to eliminate the effects of EMF using an additional magnetic sensor in TDS (on the top of the headset away from the magnetic tracer; Figure 1) and a transformation matrix between the top magnetic sensor readings and those of other magnetic sensors. The TDS training procedure was improved by recording tongue movements while the volunteer is speaking for 10 s. This procedure makes the TDS discrete commands robust against activating commands, which could occur accidentally while speaking. The TDS discrete output was updated if the past 10 classification results were the same. The TDS proportional output was obtained by averaging of outputs of the past 10 samples. These modifications made the TDS output more stable for further robotic control.

KINARM (BKIN Technologies Ltd., Kingston, ON, Canada) is an exoskeleton that can record upper limb kinematics and apply external torques to shoulder and elbow joints in the horizontal plane while providing support against gravity for both arms [52]. This device has been used in neuroscience research to quantify motor deficits and rehabilitation strategies [53].

In the integrated TDS-KA system, KINARM and TDS are connected via a serial to parallel port with a sampling rate of 200 Hz. In the TDS-KA system (see Figure 1), tongue movements were captured by a magnetic tracer on the tongue and a headset with magnetic sensors. The magnetic sensor data corresponding to the tongue position were transferred to a LabVIEW based graphical user interface (GUI) on a PC via Bluetooth low energy connection. The magnetic sensor data were further converted to either discrete commands (rest, left, right, up or down) [45] or proportional commands (a continuous number from −1 to 1) to control the exoskeleton robot via xPC target computer. The robot operator PC controlled a virtual reality display directly and the robot via a xPC target computer. The xPC target computer interacted with the exoskeleton robot via a data acquisition board (DAQ) and generated the sound queue of the task via a connected speaker.

### 2.2. Tasks

Two types of widely accepted tasks in the human-robot interaction and rehabilitation research were adopted and implemented for the TDS-KA system. These tasks were unidirectional reaching and tracking tasks.

**Figure 2:**
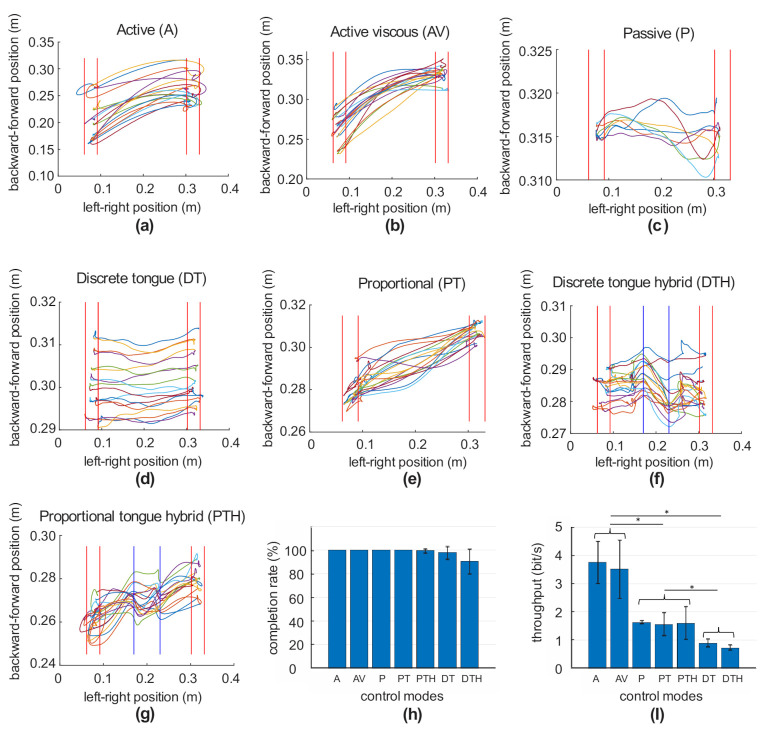
Unidirectional reaching task performance outcomes in active (A), active with viscous force field (AV), passive (P), discrete tongue control (DT), proportional tongue control (PT), discrete tongue hybrid control (DTH), and proportional tongue hybrid control (PTH) modes. Target distance is 24 cm; target width is 3 cm. (**a**–**g**) Examples of arm endpoint trajectories of one healthy subject during reaching using control modes A, AV, P, DT, PT, DTH, and PTH, respectively. The regions between the straight red lines indicate the targets. The straight blue lines in (**f**) and (**g**) indicate regions of the active range of motion without robot assistance. (**h**,**i**) Completion rate and throughput for different control modes computed across 10 healthy subjects and 18 repetitions for each. The asterisks show significant differences between control modes (*p* < 0.05, Games Howell post-hoc test).

The unidirectional reaching task was based on Fitts’ Law [54]. During each trial, the robot brought the participant’s hand to an initial position in front of the body. The participant was instructed to reach any part of a target, i.e., a band of a given width at a given distance (Figure 2a–g), in the left-right direction as quickly and accurately as possible using a specific mode. The participant’s hand needed to remain on the reached target, identified by the width of the band, for 1 s to register the attempt. Subsequently, a new target band appeared, and the subject needed to reach it as fast and accurately as possible. This was repeated 18 times by each participant.

The performance of the unidirectional reaching task was quantified using completion rate (CR) and throughput (TP). CR was defined as the percentage of trials that the participant completed within a certain period (10 s in our case). TP was calculated as follows [55]:

(1) $$TP=\frac{ID}{MT}=\frac{\log_{2}\left(\frac{D}{W}+1\right)}{MT},$$

where *ID* is the index of difficulty, *MT* is the average time to complete the movement, *D* is the distance to the target, and *W* is the target width.

Design of the tracking task was based on previous studies in upper limb rehabilitation that evaluated the accuracy of following a moving target [28,56]. The robot first brought the participant’s hand to an initial stationary target in front of the body. The target started to move in the left-right direction with a beep. The participant was asked to trace the target as accurately as possible. The position of the target as a function of time was derived from:

(2) $$x\left(t\right)\;=x_{0}+r\cdot sin\left(\omega \cdot t\right),$$

where $x_{0}$ is the initial target position in cm, $x\left(t\right)$ is the position of the moving target in cm, *r* = 12 cm is the target half-maximum left-right displacement, *ω* is an adjustable parameter that determines the rate of movements, *t* is time in milliseconds.

The performance of the tracking task was quantified using the root mean square error (*RMSE*):

(3) $$RMSE=\sqrt{\frac{1}{n}\sum_{i=1}^{n}{\left(x_{i}-{\hat{x}}_{i}\right)}^{2}},$$

where index *i* is the time sample number; *n* is the number of samples; $x_{i}$ and ${\hat{x}}_{i}$ are the left-right positions of the target and hand at time sample *i*.

### 2.3. Control Modes

We developed several modes to control movements of the upper limbs using the tongue, as well as several modes that used no assistance from the robotic system or control the arm movement without user participation. The latter modes served as baseline comparisons with the tongue-controlled modes. The modes with no or complete assistance corresponded to normal and no arm functional ability, respectively. In total, seven control modes were developed (Table 1). In discrete tongue mode (DT), the robot moved the hand in the direction of the tongue command (left, right, forward, backward or rest) with an adjustable average movement velocity such as in the passive mode (see below). The proportional tongue mode (PT) controlled arm movements by applying force to the endpoint of the arm in either the left-right or backward-forward direction [49]. The force magnitude was proportional to the difference between the tongue relative position within the range of tongue motion (e.g., between the maximal left and right positions) and the middle point of the range, where the force magnitude was zero. In active mode (A), the robot did not provide any assistance or resistance, and the user performed arm movements using their own effort. In active with viscous resistance mode (AV), the robot provided resistive force as a function of the speed of the upper limb endpoint with an adjustable gain. This mode was developed to increase resistance to movement as an additional option for rehabilitation training. In passive mode (P), the robot controlled arm movements with an adjustable average movement velocity. In the present experiments, the average velocity magnitude was *v* = 0.1 m/s.

For stroke survivors that have a limited range of motion, the rehabilitation robot should ideally assist arm movements only when needed to maximize rehabilitation outcome [57]. We developed hybrid modes that combine the active mode, engaged in the ranges of motion in which participants can control the arm themselves, with tongue-controlled modes for movement ranges in which the participants need assistance. In the hybrid modes, the tongue-driven controller automatically switches on/off when the moving arm enters/exits the range of motion where assistance is needed. We developed two versions of hybrid control modes in which the tongue controls arm motion using discrete (DTH) and proportional (PTH) controls. In these hybrid modes, the participant was instructed to use both arm and tongue control to reach targets in each task. At the same time, the viscous resistive force could be applied to the hand to make the task more challenging. For the healthy people in this study, all regions simulating limited ranges of motion were set to a fixed 6-cm interval in the middle of the task-related range of motion. For the stroke participants, this region was set based on the user’s range of motion measured before the experiment. We did not monitor possible improvements of the active range of motion in the stroke participants.

### 2.4. Experimental Protocol

All experimental procedures were consistent with the Ethical Principles for Medical Research Involving Human Subjects described in the Declaration of Helsinki and approved by the Institutional Review Board of the Georgia Institute of Technology. The participants read and signed an informed consent to participate in the study.

To demonstrate the functionality of the TDS-KA system and develop custom tasks and their feasibility for rehabilitation of the upper extremity function, we recruited 10 healthy right-handed participants (7 males and 3 females, 23–60 years; Table 2). After demonstrating safety of the developed system and all its modes of operation on the healthy volunteers, we recruited 2 female stroke survivors (32 and 58 years, Fugl-Meyer upper extremity score 35 and 13; Table 3). Since these stroke survivors had limited endurance, their testing protocol consisted of only four modes (see below).

Each healthy participant participated in one 3-h experiment session on a single day. During this session, the TDS was calibrated using standard procedures to ensure accurate performance of the device [43]. Then, the KINARM was calibrated in accordance with the system manual. Finally, the participant was asked to perform the custom-made tasks using different control modes. Each healthy participant performed the unidirectional reaching task with control modes in the following order: A, AV, P, DT, PT, DTH, and PTH (Table 1). Reaching distance was 24 cm and target width was 3 cm. Each reaching task was repeated 18 times. Each healthy participant also performed the tracking task with two different speeds (5.3 and 8 cm/s) using control modes A, AV, PT, and PTH. Each tracking task lasted 120 s. We tested the controlled modes in the same order in all subjects.

In addition, we evaluated each control mode using subjective task specific outcome measures, NASA task load index [58], and user feedback. The NASA task load index (NASA TLX) is an assessment tool for measuring subjective mental workload. It rates performance across six dimensions (mental demand, physical demand, temporal demand, performance satisfaction, frustration level, and effort) to determine an overall workload rating. For these experiments, the score was simplified to a scale of 1 to 5. We asked each healthy participant to answer the following questions for each NASA TLX dimension:

- Mental Demand: How much mental and perceptual activity was required? Was the task easy or demanding, simple or complex? 1 means low and 5 means high.
- Physical Demand: How much physical activity was required? Was the task easy or demanding, slack or strenuous? 1 means low and 5 means high.
- Temporal Demand: How much time pressure did you feel due to the pace at which the tasks or task elements occurred? Was the pace slow or rapid? 1 means low time pressure and 5 means high time pressure.
- Overall Performance: How successful were you in performing the task? How satisfied were you with your performance? 1 means not successful and 5 means successful.
- Frustration Level: How irritated, stressed, and annoyed versus content, relaxed, and complacent did you feel during the task? 1 means relaxed and 5 means stressed.
- Effort: How hard did you have to work (mentally and physically) to accomplish your level of performance? 1 means low effort and 5 means high effort.

At the end of each task and control mode combination, we recorded responses to the NASA TLX survey and noted any experiences of discomfort or additional comments from the participant.

In addition to the described experiments with healthy participants, demonstrating that the tongue control modes were safe and could be used for voluntary control of hand movements, we collected data from two female stroke survivors with upper limb movement limitations (see above). We performed the Fugl-Meyer assessment by the same assessor two weeks before the experiment, immediately before the experiment, and two weeks after the experiment began. The setup for both the stroke and healthy subject experiments were the same. However, the experimental protocol for the stroke survivors was reduced to four control modes (A, P, PT, and PTH) since these participants were tiring quickly and required more time for instructions and demonstrations, it also took longer to set up and calibrate the system with them. The same reasons prevented us from conducting the NASA TLX survey with these participants.

Stroke participant #1 had an extensive active range of motion for both elbow and shoulder joints (Fugl-Meyer upper extremity score of 35). She completed six sessions of training (3 h each, including time for instructions, demonstrations, and system setup; three sessions a week) within 2 weeks. During each session, she performed unidirectional reaching task in active (A), passive (P), and proportional tongue hybrid (PTH) modes. She also completed the tracking task in active (A) and proportional tongue (PT) modes in the left-right direction.

Stroke participant #2 had almost no active range of motion for the elbow joint and limited range of motion at the shoulder joint (Fugl-Meyer upper extremity score of 13). She also completed six sessions of training (3 h each, including time for instructions, demonstrations, and system setup; three sessions a week) within 2 weeks. However, due to the severe impairment of the most affected limb and inability of this participant to perform all the required tasks, we attempted different controlled modes in different training sessions and were able to record target tracking in the left-right directions using the PT mode in one training session only. We spent the first session helping familiarize her with the system by performing tasks in active mode with her less-impaired limb. The subsequent sessions were performed with her most impaired limb. She performed the target tracking in the left-right direction in active mode during session 2. The tracking task in the same direction was performed in proportional tongue (PT) and proportional tongue hybrid (PTH) mode in session 3. She attempted to perform the tracking task in the forward-backward direction using the active mode during session 4 (target tracking in the forward-backward directions required a much greater involvement of the most impaired elbow joint). The forward-backward tracking task was performed in proportional tongue (PT) and proportional tongue hybrid (PTH) modes during sessions 5 and 6, respectively.

We characterized the significance of differences between outcome measures of each control mode for each task in the group of healthy subjects by performing Welch’s one-way ANOVA and post-hoc Games Howell tests with the statistical significance level set to 0.05.

## 3. Results

Figure 2 (panels a–g) displays examples of hand trajectories of one healthy volunteer during repeated unilateral reaching to left and right targets (target areas are indicated by red vertical lines, distance between targets 24 cm, target width 3 cm) using control modes A, AV, P, DT, PT, DTH, and PTH, respectively. The modes A, DTH, and PTH demonstrated some overshoot of the targets, whereas the other modes did not. The hand trajectories of modes with active and tongue proportional control (A, AV, PT, and PTH) had the backward-forward displacement component in addition to the left-right component. The passive (P), discrete tongue (DT), and discrete tongue hybrid (DTH) modes had no or little backward-forward displacement component, reflecting the fact that hand trajectories in these modes were generated by the robotic system exclusively in response to commands by the robot (mode P) or the user (modes DT and DTH) to move the hand between the left and right targets. Sharp changes in hand trajectories occurred in the active region of hybrid modes DTH and PTH (the region between the straight blues lines in Figure 2f,g), indicating the switch from the tongue control to active arm control. The completion rate (CR) and throughput (TP) averaged across 10 healthy participants are shown in Figure 2h,i), respectively. All subjects were able to complete this reaching task in control modes A, AV, P, and PT (completion rate 100%). The completion rates of modes PTH, DT, and DTH were slightly smaller (range 90.6 ± 1.8% to 99.4 ± 10.5%). The highest throughput was demonstrated by the active control modes A (3.75 ± 0.75 bin/s) and AV (3.51 ± 1.04 bin/s), followed by significantly smaller throughput values of modes P (1.63 ± 0.05 bin/s), PT (1.57 ± 0.51 bin/s), and PTH (1.60 ± 0.58 bin/s) (*p* = 0.002–0.006, *n* = 10, Games Howell test) and by the smallest throughput values of modes DT (0.91 ± 0.14 bin/s) and DTH 0.73 ± 0.09 bin/s), which were statistically smaller than the throughput values of modes P, PT, and PTH (*p* = 0.002–0.049, *n* = 10, Games Howell post-hoc test; Figure 2i).

**Figure 3:**
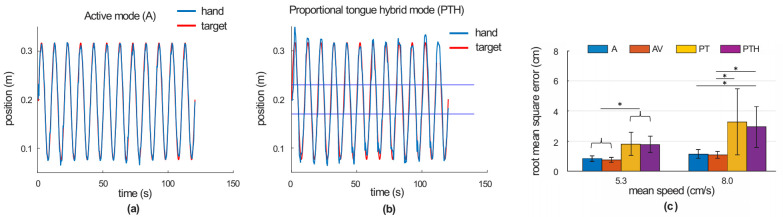
Tracking task performance outcome (RMSE) for active (A) and proportional tongue hybrid control (PTH) modes for 10 healthy participants. (**a**,**b**) Hand trajectories of a healthy person during target tracking using A and PTH modes (mean moving target speed 5.3 m/s). The blue and red lines are the subject’s hand and target positions, respectively, in the left-right direction. The region between the straight blue lines in (**b**) indicates the active range of motion without robot assistance. (**c**) The RMSE for four tested modes with two different target speeds calculated across 10 healthy subjects (mean ± S.D.). The asterisks show significant differences between control modes (*p* < 0.05, Games Howell post-hoc test).

Figure 3 (panels a and b) shows examples of hand trajectories of one healthy person during target tracking in the left-right direction using control modes A and PTH with the average moving target speed of 5.3 cm/s. Although the tongue control mode PTH demonstrated some undershoot and overshoot compared to the active arm mode A, the tracking errors were rather small for the slow tracking speed (Figure 3b). On average, the RMSE values for tongue control modes PT and PTH were statistically higher than for arm control modes A and AV for both tested tracking speeds (speed 5.3 cm/s: *p* = 0.003–0.016, *n* = 10, Games Howell post-hoc test; speed 8 cm/s: *p* = 0.011–0.056, *n* = 10, Games Howell post-hoc test; Figure 3c).

**Figure 4:**
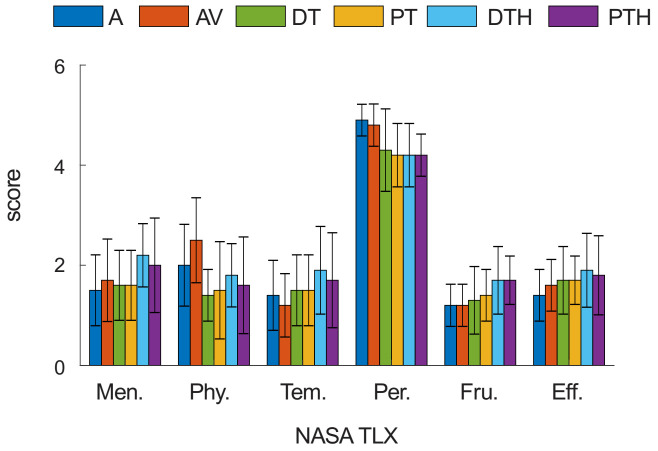
NASA task load index score (Men.: Mental demand; Phy.: Physical demand; Tem.: Temporal demand; Per.: Overall performance; Fru.: Frustration level; Eff.: Effort) for the unidirectional reaching task performed by healthy volunteers using all tested control modes. A: Active mode; AV: Active with viscous resistance mode; DT: Discrete tongue mode; PT: Proportional tongue mode; DTH: Discrete tongue hybrid mode; and PTH: Proportional tongue hybrid mode. The values are mean ± S.D. calculated across 10 healthy participants.

All participants reported a modest subjective acceptance of the system and tasks. The average scores of NASA task load index across all control modes for the mental (Men.), physical (Phy.), and temporal (Tem.) demands, as well as for frustration level (Fru.) and effort (Eff.) ranged between 1.2 and 2.5. There was no significant difference in these scores between the control modes (*p* = 0.052–0.600, *n* = 10, Welch’s test); Figure 4. These results indicate that the tasks were not very challenging for all tested modes. The overall performance (Per.) was between 4.2 to 4.9, indicating a relatively high satisfaction with the performance for all control modes. The performance of the active control mode (A) was statistically higher than that of the proportional tongue hybrid mode (PTH) (*p* = 0.008, *n* = 10, Games Howell post-hoc test; Figure 4), indicating, as could be expected, that the active and proportional tongue hybrid modes are the most and least intuitive ones, respectively.

The use of the tongue-operated upper limb robotic system and the tasks were evaluated in two chronic stroke survivors with moderate (stroke survivor #1) and severe (stroke survivor #2) paralysis—their Fugl-Meyer upper extremity assessment (FMA) scores at baseline were 35 and 13, respectively, out of maximum of 66 (Table 3). Both participants reported a modest acceptance of the system. During the 2-week period between the baseline FMA evaluation and the start of the training protocol, the FMA score of participant #1 increased from 35 to 38 and the score of participant #2 decreased from 13 to 12 (Table 3). After the six training sessions, the FMA score of participants #1 and #2 changed from 38 to 37 and from 12 to 20, respectively (Table 3).

**Figure 5:**
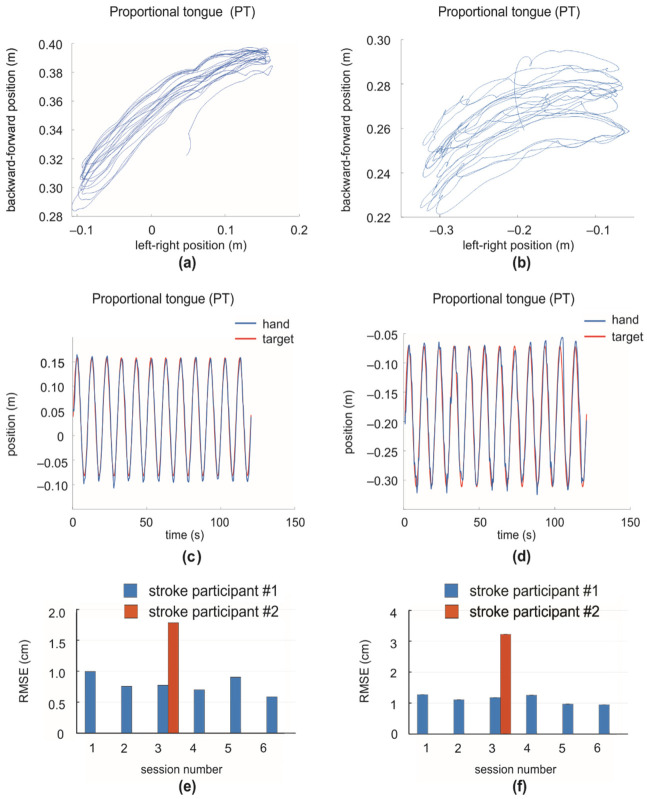
Tracking task performance of proportional tongue control (PT) for stroke participants. (**a**,**b**) Examples of hand displacements of stroke participants #1 and #2 during target tracking in the left-right directions using the proportional tongue control mode (PT); the mean speed of moving target is 5.3 cm/s. (**c**,**d**) The position of the affected hand and target as a function of time for stroke participants #1 and #2 during the target tracking using control mode (PT); the mean speed of the moving target is 5.3 cm/s. (**e**,**f**) The root mean square error (RMSE) for participants #1 and #2 during tracking a target in the left-right directions for 2 min in experimental sessions; the target speed is 5.3 cm/s in (**e**) and 8.0 cm/s in (**f**). Note that participant #2 performed this task only in session 3 (see Section 2 for details).

Panels a–b in Figure 5 show examples of hand displacements of the two stroke survivors during tracking a target moving in the left-right direction at a speed of 5.3 m/s using the proportional tongue mode. The range of backward-forward displacement for stroke participant #1 was greater indicating a larger elbow joint movement. The smoothness of hand trajectory of stroke participant #1 appeared greater than that of stroke participant #2. Panels c-d show the left-right target movement and hand position as a function of time for stroke participants #1 and #2, respectively, with a moving target speed of 5.3 cm/s. Both stroke survivors demonstrated slight overshoot of the moving target. Panels e–f in Figure 5 show the RMSE of both stroke participants for tracking sessions with target speeds of 5.3 and 8.0 cm/s. Note that participant #2 performed this task only in session 3 (see Section 2 for details). It can be observed that the accuracy of tracking the slower target is better in both participants, while the performance of stroke participant #1 is higher than in stroke participant #2. The average performance of tracking task in the proportional tongue control mode for the slower moving target (5.3 cm/s) for stroke participants #1 and #2 was 0.8 and 1.79 cm, respectively. For the fast moving target (8.0 cm/s), the mean performance was 1.12 and 3.22 cm, respectively.

## 4. Discussion

In this study, we developed a novel tongue-operated exoskeleton system for potential use in upper limb rehabilitation for people recovering from a stroke. We have evaluated the system with two custom made tasks and seven control modes of system operation in 10 healthy people and two stroke participants.

The significant performance difference between the active and tongue-operated control modes in the healthy participants indicates that the existing tongue control is still limited. One possibility could be that the participants have not fully learned how to use the tongue to control the upper limb. We need to perform a longer-term study in the future to address this issue. Another explanation for the differences between the active and tongue control modes could be the maximum force limit on the mapping between the tongue and robot movements that were set low for safety reasons.

We also noted that the performance of the proportional tongue control mode (PT) was significantly better than the performance of the discrete tongue control mode (DT) (Figure 2). This suggests that the current tongue discrete control modes are limited. For the discrete control, the tongue can only issue commands to move the hand with a fixed average velocity. In contrast, the proportional tongue control mode regulates the amount of force applied to the hand in proportion to the tongue’s relative position.

Although the performances of the active (A) and active with viscosity (AV) control modes were not significantly different, as shown in Figure 2h,i, we noted through the target overshoots that the applied viscous resistance force made the movement more accurate with less overshoot at the expense of movement speed, as shown in Figure 2a,b.

Based on the questionnaire of subjective perception of the performance (NASA TLX), the majority of the subjective performance metrics were comparable, and no significant differences were observed among the metrics, except overall performance (Figure 4). This result suggests that the perception of active and tongue control modes may be comparable. However, the physical demands tended to increase for the tongue-based operating control modes (DT, PT, DTH, PTH) compared to the active control modes (A, AV), on average. The physical demand difference is expected since in the tongue control modes, KINARM robot assists with upper limb movement. Since each participant was more familiar with the active control mode, the perceived performance for the active control modes can be higher.

The two stroke participants did not have clinically significant changes in their FMA score (should be ≥7) [59] between the baseline FMA measurement and the start of testing (Table 3). This suggests that the spontaneous recovery of upper extremity function was not observed in these participants, which was expected given that 27–62 months had passed since the ictus of stroke (Table 3). We observed that only stroke participant #2 with more severe paresis had a clinically significant improvement in FMA score after the six training sessions, even though they were not systematic (see Section 2). Despite this improvement, we cannot make a definitive conclusion about the potential benefits of the tongue-operated upper limb rehabilitation paradigm developed in this study. Additional studies with more participants are necessary.

Overall, the tongue-operated robotic system has several novel features. The system is the first to offer a way to assist in elbow and shoulder joint movements and rehabilitation via the voluntary tongue control. We added several practical and reliability improvements to the tongue drive system. Prior to this study, the TDS could not reliably provide stable control output especially in the proportional control mode.

As briefly discussed in the Introduction, the use of the tongue for the rehabilitation of arm function after a stroke can offer several unique advantages compared to other assistive robotic systems. First, the tongue is a highly innervated organ, electrical stimulation of which engages multiple cranial nerves with direct access to the brain via the brainstem and cerebellum and produces neuromodulation throughout the brain and improvements in postural, gait, cognitive, and other functions [30,60,61,62]. Therefore, the tongue motion has the potential for the neuromodulation of activity in multiple brain areas through motion-related somatosensory feedback. Second, motor equivalence, i.e., similarity of general features of movements produced by different effectors [42], may allow the tongue to guide the movement of the affected arm and strengthen its damaged cortical sensorimotor pathways without potential negative effects of making the less-impaired arm used for the same purpose more dominant [63]. Finally, the location of the tongue representation in the somatosensory cortex might be beneficial for synergistic activation of the arm representation area located in close proximity [39,64,65].

There are a number of limitations in the developed TDS-KA system in this study. The system is limited to motion in only elbow and shoulder joints in a horizontal plane, which is a substantial limitation compared to more advanced 3D arm rehabilitation robots [12]. Although the developed system demonstrated some promise for improving therapeutic outcomes in one stroke survivor, more patient data are necessary to fully evaluate the impact of rehabilitation with this system. Furthermore, we have no evidence that the tongue-controlled arm motion in the two stroke survivors enhanced the activity of the stroke-affected brain representation of the arm. The EEG brain activity recordings before and after the experiment should be investigated in the future. The tongue control currently provides only discrete and one-dimensional proportional control. By expanding the capability of TDS to 2D or 3D proportional control in the future, the system may improve its performance and enhance the recovery of arm function in more complex tasks.

## 5. Conclusions

We developed a novel tongue-operated exoskeleton system TDS-KA for potential use in upper limb rehabilitation for stroke survivors and for research in motor control. The system combines the tongue drive system and the commercially available exoskeleton KINARM. A potential advantage of the TDS-KA system is that the user can communicate the intent to move and partially control exoskeleton arms by the tongue. In turn, this could allow individuals with severe upper limb paralysis to perform rehabilitative training using their volitional control. We developed several arm reaching and tracking tasks for the TDS-KA system and evaluated the performance of these tasks in a group of healthy individuals (*n* = 10) and in two stroke survivors with upper extremity impairment. All healthy and two stroke subjects successfully performed the tasks. One stroke subject demonstrated a clinically significant improvement in Fugl-Meyer upper extremity score after practicing the tasks in six training sessions. Therefore, the TDS-KA system can accurately translate tongue commands to exoskeleton arm movements, quantify the function of the arm, and perform rehabilitation training.

## Figures and Tables

**Table 1:** The TDS-KA system control modes.

Control Mode	Description
Discrete tongue (DT)	Tongue discrete commands control robotic arm
Proportional tongue (PT)	Tongue proportional commands control robotic arm
Discrete tongue hybrid (DTH)	Combination of discrete tongue control and active control
Proportional tongue hybrid (PTH)	Combination of proportional tongue control and active control
Active (A)	No robot assistance/resistance
Active with viscous resistance (AV)	Robot provides velocity-dependent resistive load
Passive (P)	Robot controls arm movement

**Table 2:** Characteristics of healthy subjects.

Subject	Sex	Age, Years	Upper Arm Length, cm	Forearm + Hand Length, cm
1	F	26	27.6	39.5
2	M	44	28.6	47.4
3	M	23	28.5	42.9
4	F	23	27.5	35.0
5	M	24	30.6	41.9
6	M	59	31.6	50.1
7	M	23	28.6	41.0
8	F	24	28.1	37.7
9	M	24	31.0	45.6
10	M	30	30.6	42.4

**Table 3:** Stroke subject characteristics and Fugl-Meyer Assessment (FMA) for upper extremity.

Subject	Stroke Type	Sex	Affected Arm	Time since Stroke (mo)	Age (yr)	FMA at Baseline	FMA at Start	FMA at End
1	Hemorrhagic	F	Right	27	32	35/66	38/66	37/66
2	Hemorrhagic	F	Left	62	58	13/66	12/66	20/66

## Data Availability

The experimental datasets and computer code for data analysis are available from the corresponding author on reasonable request.
